# Patterns Among 754 Gamification Cases: Content Analysis for Gamification Development

**DOI:** 10.2196/11336

**Published:** 2018-11-06

**Authors:** Sungjin Park, Sangkyun Kim

**Affiliations:** 1 Management of Technology Lab Department of Industrial Engineering and Energy Research Kangwon National University Chuncheon Republic Of Korea

**Keywords:** gamification, PLEX, game mechanics, gamification correlation analysis, 4F process

## Abstract

**Background:**

Gamification is one of the techniques that applies game elements, such as game mechanics and dynamics, to a nongame context (eg, management, education, marketing, and health care). A variety of methodologies have been published for developing gamification. However, some of these are only usable by people with a certain level of gamification knowledge. People who do not have such knowledge face difficulty in using game mechanics and experiencing enjoyment. To ease their difficulties, a gamification methodology should provide directions for using game mechanics.

**Objective:**

This study aimed at collecting global gamification cases and determining patterns or differences among the collected cases.

**Methods:**

In total, 754 cases were collected based on 4F process elements, such as play type, playful user experience (PLEX)–based fun factors, and game mechanics. In addition, the collected cases were classified into 6 categories. From the data analysis, basic statistics and correlation analyses (Pearson and Kendall) were conducted.

**Results:**

According to the analysis results in PLEX-based fun factors, challenge and completion fun factors formed a large proportion among the 6 categories. In the results of the game mechanics analysis, point, leaderboard, and progress accounted for a large proportion among the 6 categories. The results of the correlation analysis showed no difference or specific patterns in game mechanics (Pearson *r*>.8, Kendall τ>.5, *P*<.05) and PLEX-based fun factors (Pearson *r*>.8, Kendall τ>.7, *P*<.05).

**Conclusions:**

On the basis of the statistical findings, this study suggests an appropriate number of PLEX-based fun factors and game mechanics. In addition, the results of this study should be used for people who do not have gamification knowledge and face difficulty using game mechanics and PLEX-based fun factors.

## Introduction

Gamification is one of the techniques that applies game elements, such as game mechanics and dynamics, to a nongame context (eg, management, education, marketing, and health care) for motivation, engagement improvement, and cooperation promotion [1,2]. Huotari and Hamari [3] defined gamification as the process of providing gameful experiences to customers and promoting customer affordance. Gamification can be applied to company operations, human resources training, marketing, and online portal to expand the loyalty, engagement, and participation of employees and customers. In 2012, Gartner Group referred to gamification as a mediator that connects technologies and humans [4]. As of 2012, gamification was classified as being in a state of *Peak of Inflated Expectation*. Gartner Group predicted that 70% of the 3000 global enterprises actively use gamification. In the schools, gamification has facilitated motivation, self-participation, and learning-effect improvement [5]. At the enterprise level, gamification was applied to a variety of fields, such as human resource management (HRM), recruitment, and incentive provision. Gamification contributes to the encouragement of employees’ sociality and significantly affects the acquisition of work-related knowledge [6].

**Figure 1:**
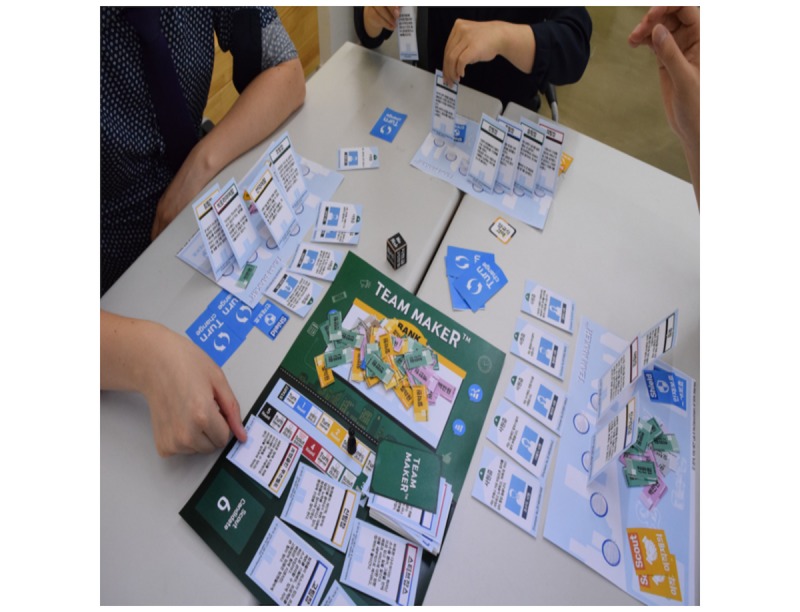
Team Maker.

**Figure 2:**
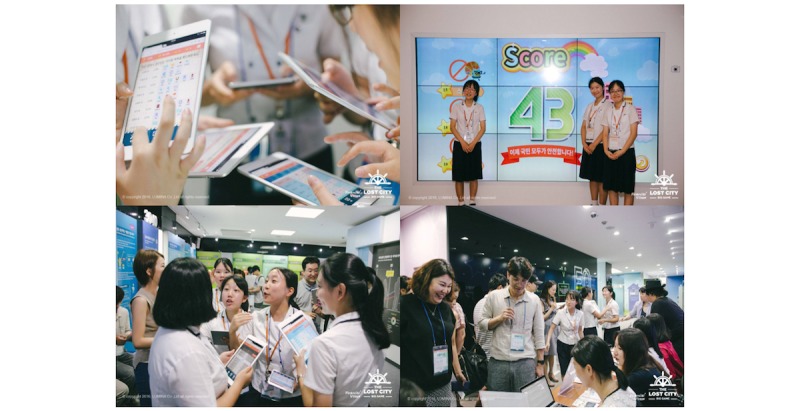
The Lost City.

Among the collected global gamification cases, some rememberable cases are summarized as follows. The IBM (International Business Machines Co) Research Center applied gamification elements, such as point, leaderboard, and feedback, to their in-house social network service (SNS) and conducted before and after comparison studies. The study results showed that the activity in the in-house SNS increased by approximately 2.5 times after the application of gamification [7]. Team Maker (Figure 1) is a case of gamification in the form of a board game developed for leadership training based on Situational Leadership II developed by Blanchard and Zigarmi [8]. Situational Leadership II is a theory indicating that team members (followers) should be classified into 4 categories according to their sociability and job-processing ability, and appropriate leadership should be applied to each type. Team Maker consists of components such as team member cards, team leader cards, and virtual currency. Team member cards are composed of cards reflecting the characteristics of members of South Korean society. Compared with general leader cards, the legend leader cards are cards comprising existing world-famous great persons; these correspond to Situational Leadership II. Before starting the game, the players select team member cards that fit the characteristics of the members of the team to which they belong. After selecting the team member cards, the team leader cards are purchased through auction during the course of the gameplay. The player that selects the team leader card that fits the team member card wins the game. The gameplay takes approximately 60 min. Figure 2 shows the case of *The Lost City*, which promotes and helps in the understanding of the financial products. The Lost City is a case of marketing gamification developed with the support of 5 major Korean banks. Each player participating in The Lost City receives a tablet personal computer to play the game, in which virtual resources are created according to game scenarios, and the team that has accumulated the largest amount of money through trading wins the game. The Lost City was designed to enable the indirect experience of real financial products, such as mortgages and loans, in the process of using financial products provided by nonplay characters in the game.

Each player should build the facilities necessary for resource supply or demand by using borrowed virtual currency and collect the virtual currency through trade among each player. The Lost City is a game played at the financial center of Yeouido, a financial city in the Republic of Korea. Kang [9] verified that The Lost City had positive effects on the understanding of financial products, understanding of how to use financial products, and financial knowledge. Moreover, Kang proposed that to maximize the effect of experience-based learning, learner’s participation promotion, and use in other educational context, gamification should be applied to learning content. With the increase in the interest in gamification, many researchers published gamification development methodology. Mora et al [10] conducted an empirical study on the gamification development methodology published from 2011 to 2015. The following are common elements of the published methodology:

- Economic (about funding and operating)
- Logic (about rule and game mechanics setting)
- Measure (about performance indication)
- Psychology (about motivating and social acting)
- Interaction (about user experience, user interface, and technology).

Bockle et al [11] published the following 4 criteria for the gamification development methodology, which allows users to develop a gamification in the order they want:

1. Purpose of adaptivity (about user’s or participant’s analysis)
2. Adaptivity criteria (about player type, context, goal setting, and level of contents)
3. Adaptive interventions (about user experience or user inerface and guideline for playing)
4. Adaptive game mechanics and dynamics (about game mechanics and dynamics).

There are many ways to develop gamification; however, the published methodologies are difficult to use as users with no experience face difficulty in using and applying game mechanics and fun experience. In addition, the number of these factors to be applied is a know-how factor. To solve some of these problems, this research team studied case studies to determine which game mechanics account for a large portion and what fun elements are used. For the case analysis, the 4F process, which is a gamification development methodology developed by Kim et al [12], was applied to analyze the game elements in collected cases. For the systematic study, the following 3 research questions were set, and 754 global gamification cases were collected and classified into 6 categories:

RQ 1: What is the distribution of global cases in terms of application category, play type, and published year?

RQ 2: What is the distribution of playful user experience (PLEX)–based fun factors in global cases, and is there a specific pattern noticeable in the applied factor among categories?

RQ 3: What is the distribution of game mechanics in global cases, and is there a specific pattern noticeable in the applied factor between among categories?

## Methods

To collect global gamification cases, Google search and the gamification website *enterprise gamification* were used and related books were referred. Only cases that can be played or whose videos or screenshots could be referenced were selected. To analyze the collected data, Microsoft Excel 2010 and R studio (Psych Package) were used. The cases were collected from October 1, 2017, to January 15, 2018. The search keywords were Enterprise Gamification (Gamification examples in Enterprise) and Gamification in (of) Marketing, Recruiting, Management, Loyalty, Engagement, and Participant.

**Figure 3:**
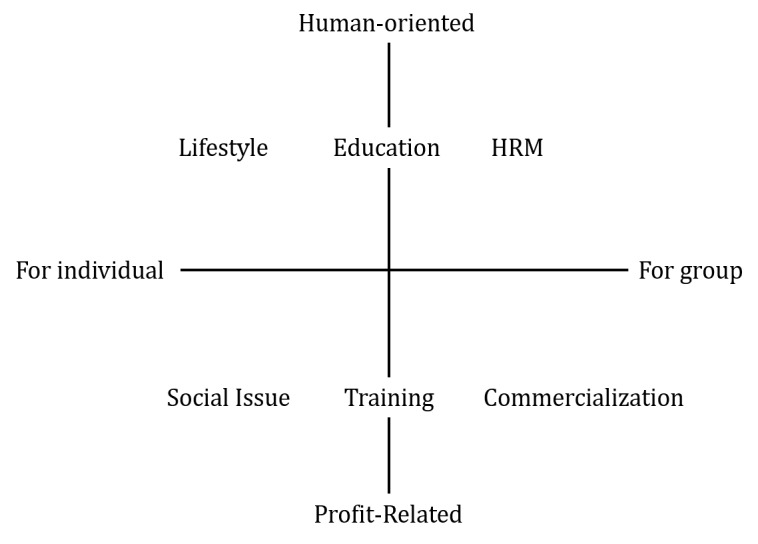
The Criteria of Six Categories. HRM: human resource management.

The collected cases were classified into the 6 categories to conduct a systematic study. There is an academical systematic study method for the paper such as the Preferred Reporting Items for Systematic Reviews and Meta-Analysis. However, no systematic study method has been proposed for general cases. An academic basis or criterion for general cases was not found. Most of the previous studies were classified according to a self-developed criterion. This study also approached in detail to categorize the collected cases according to self-developed criterion. This study was categorized from the perspectives of gamification value and player. The criteria for classification of the categories are shown in Figure 3 in which the terms *individual* and *group* were set on the x-axis from the gamification-player perspective and *human-oriented* and *profit-related* were set on the y-axis from the perspective of the gamification value. On the basis of these criteria, a total of 6 categories were derived:

- Commercialization (C): related to marketing, loyalty improvement, business management, and supply chain management
- Education (E): to obtain academic knowledge related to mathematics, science, and programming
- HRM (H): related to incentive, engagement improvement, and organization management
- Lifestyle (L): personal issue related to time management, money saving, and health care
- Social Issue (S): related to charity, car overspeeding problem, and hunger problem
- Training (T): to train technique required for specific jobs and tasks.

**Figure 4:**
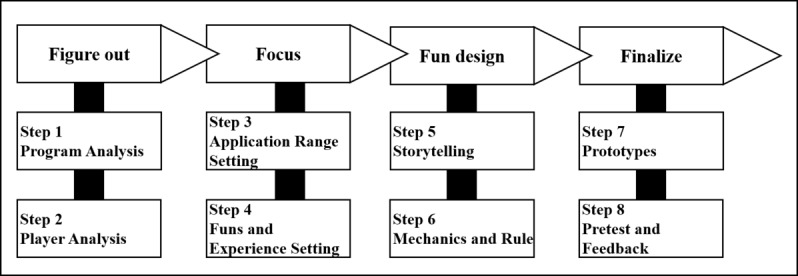
4F Process.

The case collection was based on the 4F process, as shown in Figure 4, and developed by Kim et al [12] for the development of gamification. The elements that constitute the 4F process were developed in consideration of 13 requirements for gamification development proposed by Morchhauser et al [13]. The 4F process is a development methodology to systematically manage the design process and improve the quality of gamification. The 4F process progresses in the order of Figure Out, Focus, Fun Design, and Finalize. In the Figure Out step, a targeting program or content is analyzed, and the personal identification (eg, age, gender, and favorite game genre), emotional state, and player type of participants are checked. To check the emotional state of participants in this step, the flow theory [14] for the player’s in-game emotion was applied. Player types were analyzed based on Bartle's 8 player types [15]: opportunists, planners, scientists, hackers, networkers, friends, griefers, and politicians. Player-type analysis for fundamental problem solving is essential because the preferred game or play types depend on the player types [16]. The next step, Focus, sets the goal of the gamified program or content to be developed, specifically about the part of content to be gamified, cooperation possibility of other field experts, play time, and play type.

The play type is set into software (mobile app and program), hardware (board game), and Big game or alternative reality game. The fun factors set up the experience of fun to be delivered. The fun types are applied based on the PLEX model (Table 1), which is a theory that organizes fun experienced by humans into 22 types [17]. The Fun Design step sets the option of gamification components, such as storytelling, main game mechanics, dynamics, and winning or losing elements ratios. The storytelling compresses the 12 steps of the characteristics of the story structure of Hero’s developed by Vogler and Montez [18] into 4 steps.

The game mechanics and dynamics in the 4F process were developed by referring to the framework of Mechanics, Dynamics, and Aesthetics provided by Hunicke et al [19]. Game mechanics was applied in the same or similar sense in previous studies. Kim et al [12] summarized 25 game mechanics from previous studies through empirical research. Overall, 6 large frame types were considered: reward, reward planning, avoidance, leaderboard, identification, and quest. In this study, only 18 game mechanics that can be experienced are used. The game mechanics used are listed in Table 2. The game dynamics was not defined in the form of academic rules and should be applied appropriately to match the characteristics of the player and content. The winning or losing element ratios should be set after appropriately adjusting 3 factors: player’s knowledge and skill, experience, and luck (lottery).

The Finalization step involves the completion of the prototyping of previously designed gamification and reflection through the pretester’s feedback. For the prototyping, a step-by-step manager should be determined to complete and frequently check the overall process of gamification development. After the prototyping is complete, the game should be pretested by the development team, and their feedback is supplemented. These steps are repeated to improvise the prototype’s quality, defined as good. This study applied the focus and fun design steps of the 4F process because of the following reasons. The 4F process is an academically proven model and has been applied to the proven gamification development methodology to ensure the reliability of the collected cases. In this study, the authors did their best to increase the reliability of the analysis results by using recently published gamification development methodologies.

The 4F process is a development methodology published in 2017 and has been compared with previous studies; it was thus used in this study.

**Table 1:** Description of playful user experience (PLEX) fun factors.

Playful user experience fun factors	Description
Captivation	Forgetting about one’s surroundings and flow in it
Challenge	Testing one’s ability in specific tasks
Competition	Competing himself or herself or other player (or nonplay character)
Completion	Finishing what want to do
Control	Dominating the surroundings with one’s ability
Cruelty	Causing others mental or physical pain
Discovery	Finding something new information of unknown
Eroticism	Having personal feelings for others
Exploration	Investigating a new event or situation
Expression	Manifesting oneself using item or object
Fantasy	An imagined experience in the game
Fellowship	Communicating with others and to make friend in the game
Humor	Fun, joy, joke, and gags
Nurture	Taking care of oneself or to help others to be growing
Relaxation	Relaxing and healing their mental or body in the game
Sensation	Exciting by play using 5 senses
Simulation	Testing or making something that is impossible in real world
Submission	Being part of a large group of people
Subversion	Breaking social rules or laws
Suffering	Anger, loss, and frustration
Sympathy	Sharing their emotional feelings
Thrill	Exciting derived from risk and danger

**Table 2:** Description of game mechanics used in this study.

Game mechanics	Description
Authority	The Power to control other players, town, and item ships in the game
Avatar	Displaying the player’s character visually in the game
Badge	Displaying something such as trophy and flag that player’s achievement visually
Codiscovery	Completing the quest (mission) using collective ability or collaboration with others
Competition	Competing with other players’ records or competing with their own shadow, nonplay characters
Countdown	A time when player must complete a specific quest (mission)
Leaderboard	Showing the player’s level, point, and ranks and providing the feedback
Level	Displaying player’s achievement, ability, and power to number in the game
Lottery (luck)	The gameful tool in which the winner, the course, score, and item acquisition by probability
Point	Rewarding about player’s action such as quest (mission) success
Progress (bar)	Providing real-time information about the player’s current play situation
Quest (mission)	A specific goal for the player’s growth and providing reward when solved
Real goods	Rewarding in the real world about the achievement in the game
Scaffolding	A device that reduces difficulty when faced with difficulties
Social network	Linking player to player and displaying other’s progress
Unlocking	Providing new contents and function when player clear quest (mission) or level up
Virtual goods	The item that can be purchased, acquired, and traded
Virtual money	The currency used in the game

## Results

### Case Features

Table 3 shows the results of basic statistics and classification applied to the elements of the Focus step of the 4F process. The table shows the analysis of the categories defined by the research team of this study, play types, and distribution of published year. According to the results of the basic statistics analysis and classification, HRM (148/754, 19.7%) had a relatively large proportion among the categories, followed by training (143/754, 19.0%), social issue (133/754, 17.7%), education (127/754, 16.9%), commercialization (108/754, 14.3%), and lifestyle (95/754, 12.6%).

In the results of play types, software relatively accounted for a large proportion of approximately 80.0% (603/754) among play types, followed by hardware (89/754, 11.8%) and Big game or Alternative Reality game (62/754, 8.2%). According to the results of distribution of published year, the most cases were collected in 2014 (291/754, 38.6%) and 2015 (208/754, 27.6%).

### Result of Game Mechanics and Playful User Experience–Based Fun Factors in the Cases

Tables 4 and 5 show the distribution of PLEX-based fun factors and game mechanics for each category defined in this study. Cases were recorded in the tables in order of commercialization (C), education (E), HRM (H), lifestyle (L), social issue (S), and training (T). For example, captivation of C was used 16 times out of the 108 cases (16/108, 15.0%)

The analysis results of PLEX-based fun factors are mentioned below. The factors of challenge and completion accounted for 90% in all categories. In contrast, cruelty, submission, subversion, suffering, and thrill were not found at all or only found in a few cases. In the commercialization category, competition was relatively higher than in other fun factors, and most of the PLEX-based fun factors were similarly applied compared with other categories. With respect to the education category, discovery relatively accounted for a large proportion, followed by simulation. Apart from the first 2 PLEX-based fun factors in education, the remaining fun factors were similarly applied in other categories. In the HRM category, nurture accounted for a large proportion and a specific feature pattern was not found. In the lifestyle category, expression, fellowship, relaxation, and sensation accounted for a relatively large proportion and other fun factors were similarly applied; no specific pattern was observed. In the social issue category, a specific pattern was not determined because game mechanics in social issue were applied similar to those in other categories. In the training category, discovery and simulation accounted for a large proportion, whereas no specific pattern was determined for the other fun factors.

The analysis results of game mechanics are as follows. Point, progress, and leaderboard accounted for a high proportion in all categories. In the commercialization category, the proportion of real goods was relatively higher than that in other categories. A specific pattern of game mechanics was not found in commercialization compared with other categories. In the education category, avatar, level, unlocking, and quest (mission) accounted for a large proportion. Other game mechanics were similarly applied as in other categories. In the HRM category, badge and virtual goods accounted for a large proportion. In the lifestyle category, most of the game mechanics were similarly applied. Thus, a specific pattern was not found. In the social issue category, the proportion of codiscovery was highest, and in the training category, authority, avatar, quest (mission), and unlocking accounted for a large proportion; this is similar to game mechanics distribution of the education.

**Table 3:** Case features (N=754).

Variables			n (%)
**Categories**			
	Commercialization	108 (14.3)	
	Education	127 (16.9)	
	Human resource management	148 (19.7)	
	Lifestyle	95 (12.6)	
	Social issue	133 (17.7)	
	Training	143 (19.0)	
**Play types**			
	Software	603 (80.0)	
	Hardware	89 (11.8)	
	Big game or Alternative Reality Game	62 (8.2)	
**Distribution of published year**			
	Before 2010	28 (3.8)	
	2011	22 (3.0)	
	2012	34 (4.6)	
	2013	54 (7.1)	
	2014	291 (38.6)	
	2015	208 (27.6)	
	2016	86 (11.4)	
	2017	31 (4.1)	

**Table 4:** Playful user experience (PLEX) fun factors distribution of each category.

Playful user experience	C^a^ (108 cases), n (%)	E^b^ (127 cases), n (%)	H^c^ (148 cases), n (%)	L^d^ (95 cases), n (%)	S^e^ (133 cases), n (%)	T^f^ (143 cases), n (%)
Captivation	16 (15.0)	15 (12.0)	11 (7.4)	17 (18)	16 (12.0)	19 (13.3)
Challenge	108 (100.0)	127 (100.0)	147 (99.3)	94 (99)	132 (99.2)	143 (100.0)
Competition	43 (40.0)	36 (28.3)	50 (34.0)	27 (28)	34 (26.0)	53 (37.1)
Completion	101 (94.0)	127 (100.0)	148 (100.0)	94 (99)	132 (99.2)	143 (100.0)
Control	6 (6.0)	17 (13.4)	19 (13.0)	12 (13)	16 (12.0)	27 (19.0)
Cruelty	0 (0.0)	0 (0.0)	0 (0.0)	0 (0)	0 (0.0)	1 (1.0)
Discovery	63 (58.3)	98 (77.2)	67 (45.3)	48 (51)	74 (56.0)	90 (63.0)
Eroticism	0 (0.0)	0 (0.0)	0 (0.0)	1 (1)	2 (2.0)	0 (0.0)
Exploration	38 (35.2)	35 (29.0)	13 (9.0)	18 (19)	28 (21.0)	31 (22.0)
Expression	58 (54.0)	47 (37.0)	65 (44.0)	62 (65)	69 (52.0)	74 (52.0)
Fantasy	24 (22.2)	44 (35.0)	13 (9.0)	21 (22)	20 (15.0)	33 (23.1)
Fellowship	48 (44.4)	36 (28.3)	66 (45.0)	52 (55)	64 (48.1)	52 (36.4)
Humor	11 (10.2)	3 (2.4)	7 (5.0)	11 (12)	10 (8.0)	4 (3.0)
Nurture	32 (30.0)	53 (42.0)	75 (51.0)	46 (48)	60 (45.1)	55 (38.5)
Relaxation	50 (46.3)	33 (26.0)	53 (36.0)	46 (48)	62 (47.0)	33 (23.1)
Sensation	25 (23.1)	46 (36.2)	27 (18.2)	45 (47)	31 (23.3)	53 (37.1)
Simulation	29 (27.0)	86 (68.0)	45 (30.4)	30 (32)	63 (47.4)	107 (75.0)
Submission	1 (1.0)	1 (1.0)	0 (0.0)	1 (1)	2 (2.0)	2 (1.4)
Subversion	0 (0.0)	0 (0.0)	0 (0.0)	0 (0)	0 (0.0)	0 (0.0)
Suffering	1 (1.0)	0 (0.0)	0 (0.0)	0 (0)	0 (0.0)	4 (3.0)
Sympathy	28 (26.0)	14 (11.0)	37 (25.0)	28 (29)	65 (49.0)	37 (26.0)
Thrill	3 (3.0)	1 (1.0)	0 (0.0)	4 (4)	4 (3.0)	6 (4.2)

^a^C: commercialization.

^b^E: education.

^c^H: human resource management.

^d^L: lifestyle.

^e^S: social issue.

^f^T: training.

**Table 5:** Game mechanics distribution of each category.

Mechanics	C^a^ (108 cases), n (%)	E^b^ (127 cases), n (%)	H^c^ (148 cases), n (%)	L^d^ (95 cases), n (%)	S^e^ (133 cases), n (%)	T^f^ (143 cases), n (%)
Authority	20 (19.0)	33 (26.0)	30 (20.3)	21 (22)	36 (27.0)	44 (31.0)
Avatar	41 (38.0)	76 (60.0)	65 (44.0)	38 (40)	33 (25.0)	84 (59.0)
Badge	78 (72.2)	73 (57.5)	125 (84.5)	73 (77)	84 (63.1)	79 (55.2)
Codiscovery	54 (50.0)	56 (44.1)	54 (36.5)	44 (46)	76 (57.1)	64 (45.0)
Competition	42 (39.0)	38 (30.0)	50 (34.0)	26 (27)	34 (26.0)	55 (38.5)
Countdown	29 (27.0)	49 (39.0)	27 (18.2)	32 (34)	45 (34.0)	58 (41.0)
Leaderboard	102 (94.4)	124 (98.0)	146 (99.0)	94 (99)	128 (96.2)	140 (98.0)
Level	20 (19.0)	63 (50.0)	32 (22.0)	28 (29)	19 (14.3)	46 (32.2)
Lottery (luck)	30 (28.0)	31 (24.4)	19 (13.0)	24 (25)	31 (23.3)	35 (24.5)
Point	87 (81.0)	119 (94.0)	144 (97.3)	84 (88)	112 (84.2)	132 (92.3)
Progress	105 (97.2)	126 (99.2)	146 (99.0)	94 (99)	132 (99.2)	143 (100.0)
Quest (mission)	48 (44.4)	90 (71.0)	53 (36.0)	48 (51)	61 (46.0)	82 (57.3)
Real goods	38 (35.2)	6 (5.0)	16 (11.0)	12 (13)	40 (30.1)	10 (7.0)
Social network	35 (32.4)	14 (11.0)	27 (18.2)	16 (17)	22 (17.6)	15 (10.5)
Scaffolding	20 (19.0)	46 (36.4)	20 (14.0)	16 (17)	28 (21.6)	36 (25.2)
Unlocking	21 (19.4)	45 (35.4)	24 (16.2)	22 (22)	29 (22.0)	50 (35.0)
Virtual goods	59 (55.0)	76 (60.0)	90 (61.0)	45 (45)	59 (44.4)	72 (50.3)
Virtual money	14 (13.0)	31 (24.4)	34 (23.0)	19 (19)	30 (23.0)	25 (17.5)

^a^C: commercialization.

^b^E: education.

^c^H: human resource management.

^d^L: lifestyle.

^e^S: social issue.

^f^T: training.

### Correlation of Game Mechanics and Playful User Experience Fun Factors Distribution

The results of the correlation analysis are summarized in Tables 6 and 7. In this study, Pearson and Kendall correlation coefficients were used in the correlation analysis. Both parametric and nonparametric analyses should be conducted to check the statistical significance [20,21]. Pearson correlation coefficient is a type of parametric correlation analysis, and Kendall correlation analysis is a type of nonparametric correlation analysis. This study has tried to ensure the reliability of the results through 2 correlation methods.

According to Gustavo et al [22], the standard Pearson and Kendall correlation coefficients are as follows:

- small effect: τ=.20 is approximately equal to *r*=.30
- medium effect: τ=.34 is approximately equal to *r*=.50
- large effect: τ=.50 is approximately equal to *r*=.70.

The results of the game mechanics correlation for each category are as follows. There is a statistically large correlation among each category (Tables 6 and 7). The category of commercialization and other categories are significantly correlated with education (*r*=.822, *P*<.001, τ=.518, *P*=.003), HRM (*r*=.949, *P*<.001, τ=.598, *P*<.001), lifestyle (*r*=.951, *P*<.001, τ=.678, *P*<.001), social issue (*r*=.956, *P*<.001, τ=.713, *P*<.001), and training (*r*=.877, *P*<.001, τ=.607 *P*<.001). The categories of HRM (*r*=.882, *P*<.001, τ=.682, *P*<.001), lifestyle (*r*=.927, *P*<.001, τ=.801, *P*<.001), social issue (*r*=.846, *P*<.001, τ=.507, *P*=.003), and training (*r*=.972, *P*<.001, τ=.862, *P*<.001) are significantly correlated with education. The HRM category is significantly correlated with lifestyle (*r*=.969, *P*<.001, τ=.742, *P*<.001), social issue (*r*=.920, *P*<.001, τ=.559, *P*=.001), and training (*r*=.913, *P*<.001, τ=.743, *P*<.001). The categories of lifestyle and other categories are significantly correlated with social issue (*r*=.958, *P*<.001, τ=.665, *P*<.001) and training (*r*=.952, *P*<.001, τ=.849, *P*<.001). The categories of social issue and training are significantly correlated by *r*=.897 (*P*<.001), τ=.608 (*P*<.001).

The results of the correlation analysis of PLEX fun factors are as follows. There is a statistically large correlation among each category (Tables 8 and 9). The category of commercialization and other categories are significantly correlated with education (*r*=.898, *P*<.001, τ=.735, *P*<.001), HRM (*r*=.951, *P*<.001, τ=.828, *P*<.001), lifestyle (*r*=.956, *P*<.001, τ=.841, *P*<.001), social issue (*r*=.953, *P*<.001, τ=.841, *P*<.001), and training (*r*=.905, *P*<.001, τ=.773, *P*<.001). The categories of education and other categories are significantly correlated with HRM (*r*=.893, *P*<.001, τ=.774, *P*<.001), lifestyle (*r*=.88, *P*<.001, τ=.787 *P*<.001), social issue (*r*=.9, *P*<.001, τ=.77, *P*<.001), and training (*r*=.975, *P*<.001, τ=.871, *P*<.001). The categories of lifestyle (*r*=.961, *P*<.001, τ=.841, *P*<.001), social issue (*r*=.97, *P*<.001, τ=.832, *P*<.001), and training (*r*=.921, *P*<.001, τ=.794, *P*<.001) are significantly correlated with HRM. Lifestyle is significantly correlated with social issue (*r*=.959, *P*<.001, τ=.889, *P*<.001) and training (*r*=.906, *P*<.001, τ=.803, *P*<.001). The categories of social issue and training are significantly correlated by *r*=.935 (*P*<.001), τ=.812 (*P*<.001).

### Result of Number of Game Mechanics and Playful User Experience Fun Factors Combination

Tables 10 and 11 list the results of the number of game mechanics and PLEX fun factors used through collected cases. A total of 6 PLEX fun factors were used on average. The minimum number of fun factors was 2 and the maximum number was 18. Moreover, 4 to 8 PLEX fun factors accounted for 71% of the overall categories. The average number of game mechanics was 8. The minimum number of game mechanics was 3 and the maximum number was 18. In addition, 5 to 9 game mechanics accounted for 66% of the overall categories.

**Table 6:** Pearson correlation results of game mechanics.

Game mechanics	Pearson correlation				
	C^a^	E^b^	H^c^	L^d^	S^e^
E	.822^f^	—	—	—	—
H	.949^f^	.882^f^	—	—	—
L	.951^f^	.927^f^	.968^f^	—	—
S	.956^f^	.846^f^	.919^f^	.958^f^	—
T^g^	.877^f^	.972^f^	.913^f^	.952^f^	.897^f^

^a^C: commercialization.

^b^E: education.

^c^H: human resource management.

^d^L: lifestyle.

^e^S: social issue.

^f^*P*<.001.

^g^T: training.

**Table 7:** Kendall correlation results of game mechanics.

Game mechanics	Kendall correlation				
	C^b^	E^c^	H^d^	L^e^	S^f^
E	.518^f^	—	—	—	—
H	.598^g^	.682^g^	—	—	—
L	678^g^	.801^g^	.742^g^	—	—
S	.713^g^	.507^f^	.559^h^	.665^g^	—
T^g^	.607^g^	.862^g^	.743^g^	.849^g^	.608^g^

^a^C: commercialization.

^b^E: education.

^c^H: human resource management.

^d^L: lifestyle.

^e^S: social issue.

^f^*P*=.003.

^g^*P*<.001.

^h^*P*=.001.

^g^T: training.

**Table 8:** Pearson correlation results of playful user experience (PLEX) fun factors.

Game mechanics	Pearson correlation				
	C^a^	E^b^	H^c^	L^d^	S^e^
E	.898^f^	—	—	—	—
H	.951^f^	.893^f^	—	—	—
L	.956^f^	.880^f^	.961^f^	—	—
S	.953^f^	.900^f^	.970^f^	.959^f^	—
T^g^	.905^f^	.975^f^	.921^f^	.906^f^	.935^f^

^a^C: commercialization.

^b^E: education.

^c^H: human resource management.

^d^L: lifestyle.

^e^S: social issue.

^f^*P*<.001.

^g^T: training.

**Table 9:** Kendall correlation results of playful user experience (PLEX) fun factors.

Game mechanics	Kendall correlation				
	C^a^	E^b^	H^c^	L^d^	S^e^
E	.735^f^	—	—	—	—
H	.828^f^	.774^f^	—	—	—
L	.841^f^	.787^f^	.84^f^	—	—
S	.841^f^	.770^f^	.832^f^	.889^f^	—
T^g^	.773^f^	.871^f^	.794^f^	.803^f^	.812^f^

^a^C: commercialization.

^b^E: education.

^c^H: human resource management.

^d^L: lifestyle.

^e^S: social issue.

^f^*P*<.001.

^g^T: training.

**Table 10:** Average, minimum, and maximum number of applied game mechanics and playful user experience (PLEX) fun factors.

Factors and mechanics		n	
**Playful user experience fun factors**			
	Average		6
	Minimum		2
	Maximum		18
**Game mechanics**			
	Average		8
	Minimum		3
	Maximum		18

**Table 11:** Number of applied Game mechanics and playful user experience (PLEX) fun factors.

Distribution		n (%)	
**Number of playful user experience** **fun factors**			
	2		19 (2.6)
	3		59 (8.0)
	4		91 (12.1)
	5		145 (19.2)
	6		129 (17.1)
	7		88 (12.0)
	8		81 (10.8)
	9		46 (6.1)
	10		31 (4.1)
	11		31 (4.1)
	12		15 (2.0)
	13		9 (1.2)
	14		5 (1.0)
	15		1 (0.3)
	16		3 (0.3)
	18		1 (0.3)
	Total		754 (100.0)
**Number of game mechanics**			
	2		14 (2.0)
	3		50 (7.0)
	4		81 (11.0)
	5		115 (15.2)
	6		116 (15.4)
	7		103 (14.0)
	8		84 (11.1)
	9		61 (8.1)
	10		55 (7.3)
	11		29 (4.0)
	12		18 (2.4)
	13		10 (1.3)
	14		13 (2.0)
	15		1 (0.3)
	16		3 (0.3)
	18		1 (0.3)
	Total		754 (100.0)

## Discussion

### Principal Findings

The purpose of this study was to collect 754 gamification cases and derive their implication through an empirical study and a statistical data analysis. The 4F process was applied for analyzing gamification elements. Table 12 summarizes the answers to the 3 research questions. The results of this study would help people who have difficulty in developing gamification. When developing gamification, we expect that the results of this study will have a positive impact on people who have difficulty using game mechanics and experiencing fun.

**Table 12:** Summary of results.

Research question and question keywords		Result
**1**		
	Categories	Category classification: 6 categories
	Play types	Play types: software (80%), hardware (12%), and Big game or ARG^a^ (8%)
	Published year	High proportion of published year: 2014 and 2015
**2**		
	PLEX^b^ fun factors distribution	High proportion of PLEX fun factors: challenge and completion; low proportion of PLEX fun factors: cruelty, submission, subversion, suffering, and thrill
	Correlation each category	Correlation analysis: distribution of PLEX fun factors was similar in each category (a positive correlation for each category)
**3**		
	Game mechanics distribution	High proportion of game mechanics: point, progress, and leaderboard; proportion of game mechanics was similarly distributed and used
	Correlation each category	Correlation analysis: distribution of game mechanics was similar in each category (a positive correlation for each category)

^a^ARG: alternative reality game.

^b^PLEX: playful user experience.

### Discussion of Research Question 1

Among the 3 types of gameplay, software accounted for 80% of overall cases. It is related to recent advances in information technology such as smartphones, smartpads, and improvement of internet environment. As technology advances, because access to software type of gamification has been strengthened, the player is possible to play the variety of type of gamification contents without time and space constraints. Furthermore, software type of gamification is used to secure marketing and potential customer and their loyalty, and it is possible to solve the inconvenience of accessing services such as online banking system and stock trading system.

Baptista and Oliveira [23] applied gamification to a software type of mobile banking system to improve its usability and accessibility to customers. For validating the results of their study, a structural equation was used. According to the results of their study, a software type of gamification was possible to improve customers’ engagement, accessibility, and usability. Baptista and Oliveira [23] suggested using gamification for changing customers’ behavior through gaming experience.

According to the results of this study, the largest number of gamification cases are published in 2014 and 2015. It is assumed that the collected cases are maintained through continuous updating. However, this does not imply that new cases have not been published. Global gamification cases have increased steadily since gamification was activated, with a significant increase observed in 2014 and 2015, and these cases are being kept. Dicheva et al [24] conducted an empirical research of an advanced study on gamification in education. The number of education gamification cases began increasing from 2013, and the distribution of overall cases was similar to that obtained in this study. Additional studies should be conducted to infer clear conclusions about the year of distribution for published cases.

### Discussion of Research Question 2

In the analysis results of PLEX fun factors from among the 6 categories defined in this study, challenge, completion, and competition constituted a large proportion of overall categories. The distribution of PLEX fun factors in this study resembles that of Kim’s study [25]. The reason why the fun factors of challenge and completion accounted for a large proportion is assumed to be related to the winning condition. The structure of gamification is also designed to be similar to that of the game. Players must challenge and solve problems to win the game. Gamification also provides a resolvable problem such as a game for players. In the process of problem solving, the fun factors of challenge and completion are added. For this reason, both these fun factors accounted for a large proportion among the fun factors. The fun factor of competition was used assuming that it will allow more players to participate. On the other hand, the fun factors of cruelty, submission, subversion, suffering, and thrill were either found in a few cases or not found in the collected cases. These 5 fun factors were found to be very difficult to implement and apply within game-based content.

The correlation analysis results of PLEX fun factors showed similar patterns among the 6 categories. This means that there is no particularly preferred pattern according to the application field of the PLEX fun factors. Having no particularly preferred pattern among the PLEX fun factors implies that there was no difference among the fun factors. The correlation analysis results can be shown as the grounds for the use of suitable PLEX fun factors regardless of the applied target. However, reckless use of fun factors is harmful to the quality of gamification. Depending on the results of this study, it is recommended to use 4 to 8 PLEX fun factors.

### Discussion of Research Question 3

In the analysis results of game mechanics among the 6 categories defined in this study, points, progress, and leaderboard constituted a large proportion of the overall categories. The distribution of game mechanics in this study resembles that of Dicheva et al’s study [24]. In most cases, points have been shown to be used as concept of reward. Progress and leaderboard have been using similar concepts in most cases. However, both these game mechanics are of a different nature and should be analyzed separately. On the other hand, badge, a Point, Badge, and Level system [26], found its utilization to be lower than points and leaderboard. It is assumed to have been caused by regional or cultural differences. In North America, many people are found collecting badges from local universities. In Asia, badge collection is quite strange. Due to these regional or cultural differences, it is assumed that the use of badge is relatively low. Specially, among the 6 categories of social issues, the game mechanics of codiscovery was found to be relatively higher than that in other categories. It is important to address social issues, but it can be thought of as using collective intelligence.

In the correlation analysis results of game mechanics, the correlation among the 6 categories was significant. This indicates that most categories are based on similar game mechanics. However, reckless use, such as of PLEX fun factors, can harm players. This study recommends using 5 to 9 game mechanics when exploring game mechanics. A designer or developer would like to use these many game mechanics during the gamification development process.

### Limitations and Future Direction

The limitations of this study are as follows:

- Criteria for case classification without Mutually Exclusive and Completely Exhaustive (MECE)
- Absence of a differentiated method of data analysis
- Validity and reliability problems caused by applying a single gamification development methodology
- Limits of the restricted search range centered on the Google search engine and *enterprise gamification* websites.

Before conducting this study, criteria were set to classify the cases. However, the category could not fully classify the cases because MECE was not applied. Using MECE and a proven taxonomy to analyze the cases based on academic grounds, more relevant study results must be obtained.

Recent developments in big data have begun to refine data analytics. This study only performed basic statistical analyses on the correlation between PLEX fun factors and game mechanics. However, further studies should be conducted considering not only the number used in the data analysis techniques but also the relevant factors such as player type. For this, data should be collected more systematically, and analysis techniques should be applied accordingly to conduct further studies.

This study only used the 4F process to analyze the collected cases. Therefore, analytical factors from different perspectives should be obtained. Researchers in related fields are constantly developing methodologies to develop systematic gamification such as 6D process [27] and the 13 requirements for gamification development [13]. In addition, player-type classification procedures should be added through data analysis. Examples of representative player type classifications include Bartle’s 8 player types [15], BrainHex [28], and Octalysis [29].

The cases used in this study were collected only through Google search and *enterprise gamification* Web page. However, future study should be conducted on a wider range of cases, including cases analyzed in previous papers. Future study should be conducted through diverse case collection for verifying the reliability and feasibility of study results.

## Abbreviations

HRM: human resource management
MECE: Mutually Exclusive and Completely Exhaustive
PLEX: playful user experience
SNS: social network service
